# Purification, crystallization and X-ray structures of the two manganese superoxide dismutases from *Caenorhabditis elegans*

**DOI:** 10.1107/S1744309108037056

**Published:** 2008-11-28

**Authors:** Chi H. Trinh, Thérèse Hunter, Emma E. Stewart, Simon E. V. Phillips, Gary J. Hunter

**Affiliations:** a Astbury Centre for Structural Molecular Biology, Institute of Molecular and Cellular Biology, University of Leeds, Leeds LS2 9JT, England; b Department of Physiology and Biochemistry, University of Malta, Msida, MSD 2080, Malta

**Keywords:** manganese superoxide dismutases, *Caenorhabditis elegans*

## Abstract

*Caenorhabditis elegans* expresses two manganese superoxide dismutase enzymes (MnSOD-2 and MnSOD-3) that are targeted to the mitochondrion. MnSOD-2 is constitutively expressed, while synthesis of MnSOD-3 is inducible. The structures of these two mononuclear metalloenzymes have been determined to 1.8 and 1.7 Å resolution, respectively. Pink crystals formed in space group *P*4_1_2_1_2 for each, with unit-cell parameters *a* = *b* = 81.0, *c* = 137.4 Å for MnSOD-2 and *a* = *b* = 81.8, *c* = 136.0 Å for MnSOD-3. The final structure of MnSOD-3 was refined to *R* = 21.6% and *R*_free_ = 26.2% at 293 K, and *R* = 18.9% and *R*_free_ = 22.6% at 100 K, while that of MnSOD-2 was refined to *R* = 16.9% and *R*_free_ = 20.1% at 100 K. The asymmetric unit cell is comprised of two subunits. The resulting structures are very similar to that of human MnSOD and form a tetramer corresponding to a dimer of dimers. The subunit interface between dimers is comprised of two four-helix bundles that stabilize the biologically significant homotetramer.

## Abstract 2

Two manganese superoxide dismutase enzymes isolated from the eukaryote *C. elegans* have been characterized and their structures determined. The closely related structures reveal a striking similarity to manganese superoxide dismutase found in humans.

## 1. Introduction   

The superoxide dismutases (SODs; EC 1.15.11) are ubiquitous metalloproteins whose purpose is to detoxify the highly reactive superoxide anion (O_2_^−^) by its dismutation into oxygen and hydrogen peroxide (McCord & Fridovich, 1969 ▶). The superoxide anions would otherwise react with cell constituents, causing oxidative damage to macromolecules including lipids, DNA and proteins (Halliwell & Gutteridge, 1985 ▶). Since hydrogen peroxide, a product of the dismutation, is itself toxic to cells, the presence of SOD is intimately linked with that of catalase and peroxidase. Many organisms contain more than one type of SOD, distinguishable by their metal cofactor and subcellular location. Most eukaryotes, such as *Caenorhabditis elegans*, express both Cu/ZnSOD and MnSOD (Fridovich, 1975 ▶). Homologous FeSOD and MnSOD are expressed in prokaryotes (Bannister *et al.*, 1987 ▶). *C. elegans* is a free-living nematode that lives in temperate soil environments. It serves as an interesting model to investigate SOD since it encodes five *sod* genes producing two cytosolic Cu/ZnSODs (SOD-1 and SOD-5), one extracellular Cu/ZnSOD (SOD-4) and two MnSODs (SOD-2 and SOD-3; designated MnSOD-2 and MnSOD-3, respectively) (*C. elegans* Genome Consortium, 1998 ▶). MnSOD-2 and MnSOD-3 are homologous proteins and are both synthesized as precursors with N-terminal mitochondrial targeting signals (Hunter *et al.*, 1997 ▶). Extensive studies have revealed differential expression patterns, with MnSOD-2 emerging as the major constitutive MnSOD. MnSOD-3 is associated with diapause and its expression is induced in the long-lived dauer stage (Honda & Honda, 1999 ▶; Jones *et al.*, 2001 ▶). Dissection of the insulin/IGF signalling pathway has identified *sod-3* as a significant target for the DAF-16/FOXO transcription factor. Inhibition of the insulin/IGF signalling pathway results in elevated expression of *sod-3* and an extension in lifespan (Murphy *et al.*, 2003 ▶; Lee *et al.*, 2003 ▶; Dong *et al.*, 2007 ▶). Recently, it has been suggested that these MnSODs function in the insulin/IGF signalling pathway as physiological redox modulators rather than antioxidants (Honda *et al.*, 2008 ▶). In a complementary approach to the study of differences in expression and cellular roles of the SODs of *C. elegans*, we are investigating the differences between the proteins themselves. Here we present the structures of the two MnSODs which are strikingly similar to that from human (Hearn *et al.*, 2003 ▶).

## 2. Materials and methods   

### 2.1. Protein expression and purification   

A polymerase chain reaction amplicon encoding the presumed mature MnSOD-2 and MnSOD-3 proteins was cloned into the expression vector pTrc-99A as described previously (Hunter *et al.*, 1997 ▶). The integrity of the promoter and SOD sequences was confirmed by DNA sequencing. Cultures (500 ml) of *Escherichia coli* strain OX326A (*ΔsodA*, *ΔsodB*) (Steinman, 1992 ▶) harbouring the required expression clone were grown in 2×YT rich media (16 g l^−1^ tryptone, 10 g l^−1^ yeast extract and 5 g l^−1^ NaCl) supplemented with ampicillin (100 µg ml^−1^) and manganese sulfate (50 µ*M*) at 303 K in a shaking incubator. When the cell density reached an OD_600_ of 0.4, protein expression was induced by the addition of isopropyl β-d-1-thiogalactopyranoside to a final concentration of 1 m*M*. After 4 h cells were harvested by centrifugation and resuspended in phosphate-buffered saline pH 7.8 containing 0.03% sodium dodecyl sulfate and 0.1% Triton X-100. Cells were lysed by passage through a French pressure cell at 110 MPa (Aminco). Ultrasonic disruption was used to reduce the viscosity of the resulting solution. Clarification was performed by centrifugation (10 000*g*, 20 min) followed by filtration through a 0.45 µm Nalgene syringe filter.

Protein extracts were applied onto nickel-charged 5 ml Hi-Trap columns (GE Healthcare) for metal-chelation affinity chromatography (MCAC). Columns were washed after loading with 20 m*M* phosphate buffer pH 7.8 containing 500 m*M* NaCl. Imidazole in the same phosphate buffer was used first to wash the columns (2 m*M* for MnSOD-2, 1 m*M* for MnSOD-3) and then to elute the SOD proteins (50 m*M* for MnSOD-2, 20 m*M* for MnSOD-3). The collected SOD-containing fractions were dialysed against 10 m*M* Tris–HCl pH 7.8 buffer and subjected to a second round of MCAC purification as above. Ion exchange using CM-52 pre-equilibrated with 20 m*M* bis-Tris–HCl pH 6 was used to further purify MnSOD-3. Addition of bis-Tris–HCl to the MnSOD-3 sample resulted in the precipitation of a high-molecular-weight (∼60 000 Da) contaminant protein. This was removed by centrifugation before the MnSOD-3 was incubated with the CM-52. After binding, the CM-52 was washed in batch several times with 10 m*M* bis-Tris–HCl pH 6 and MnSOD-3 was then eluted using 10 m*M* Tris–HCl pH 8 containing 100 m*M* NaCl. Combined eluates were concentrated prior to application onto a gel-filtration column. Both MnSOD-2 and MnSOD-3 were further purified on a Sephacryl S-200 gel-filtration column using 10 m*M* Tris–HCl pH 8.0 and 0.15 *M* NaCl at a flow rate of 0.8 ml min^−1^. SOD-containing fractions were pooled and concentrated by centrifugal ultrafiltration (Millipore). All purifications were followed and assessed by both SDS–PAGE (Laemmli, 1970 ▶) and SOD-stained native PAGE (Beauchamp & Fridovich, 1971 ▶). Purified MnSOD-2 and MnSOD-3 were stored at concentrations of 6.6 and 8.0 mg ml^−1^, respectively, at 193 K in 10 m*M* Tris–HCl pH 7.8.

### 2.2. Characterization and crystallization   

The spectrophotometric assay for SOD used the cytochrome *c* method (McCord & Fridovich, 1969 ▶; Ysebaert-Vanneste & Vanneste, 1980 ▶) and protein estimation used the bicinchoninic acid assay (Smith *et al.*, 1985 ▶). Metal analysis by inductively coupled plasma-sector field mass spectrometry was performed by ALS Analytica AB, Sweden. MnSOD samples in 40 m*M* ammonium acetate were treated with methanol and formic acid and analysed on an electrospray mass spectrometer (Platform II and Q-TOF) to estimate molecular mass.

The hanging-drop vapour-diffusion method was used with drops consisting of 1 µl protein and 1 µl reservoir solution equilibrated at 291 K. The optimal reservoir solution (500 µl) for MnSOD-2 (3 mg ml^−1^) contained 0.1 *M* Bicine pH 9.2 and 3.0 *M* ammonium sulfate and that for MnSOD-3 (8 mg ml^−1^) contained 0.1 *M* Bicine pH 9.2 and 2.7 *M* ammonium sulfate. Crystals typically grew to 350 × 300 × 300 µm and were transferred to a cryoprotectant solution containing sodium malonate (1.7 *M* final concentration) before mounting in loops for data collection. Crystals were additionally mounted in a glass capillary for data collection at 293 K.

### 2.3. Data collection and processing   

Data for MnSOD-3 were recorded at 293 K with an R-AXIS IV^++^ image-plate detector mounted on a Rigaku RU-H3R rotating-anode X-ray generator and were integrated using *MOSFLM* (Leslie, 1999 ▶) to 1.77 Å resolution. Subsequently, X-ray diffraction data for both MnSOD-2 and MnSOD-3 were recorded at 100 K on station I03 at Diamond Light Source using an ADSC Quantum 315 charge-coupled device (CCD) detector. The Diamond Light Source MnSOD-2 and MnSOD-3 data were integrated to 1.8 and 1.7 Å resolution, respectively, using the program *MOSFLM*. Data reduction and subsequent calculations were carried out using the *CCP*4 program suite (Collaborative Computational Project, Number 4, 1994 ▶). Both MnSOD-2 and MnSOD-3 crystals belonged to space group *P*4_1_2_1_2, with unit-cell parameters *a* = *b* = 81.0, *c* = 137.4 Å for MnSOD-2 and *a* = *b* = 81.8, *c* = 136.0 Å for MnSOD-3. There are two subunits of MnSOD-2 and MnSOD-3 per asymmetric unit (Table 1 ▶).

### 2.4. Structure solution and refinements   

**Figure 1:**
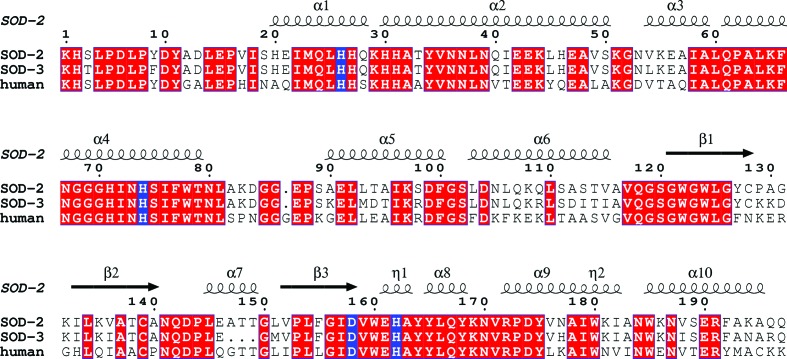
Protein-sequence alignment of the three manganese superoxide dismutases MnSOD-2 (SOD-2), MnSOD-3 (SOD-3) and human MnSOD (Borgstahl *et al.*, 1992 ▶) with the secondary-structure elements of MnSOD-2 superposed. The two domains extend between residues 1 and 81 (N-domain), forming an α-hairpin with a turn between α2 and α3, and residues 90 and 197 (C-domain). Helix α7 is absent in MnSOD-3 owing to the deletion of residues 147–149. The alignment figure was made using the program *ESPript* (Gouet *et al.*, 1999 ▶). Red boxes indicate strictly conserved residues. The helices labelled η1 and η2 represent 3_10_-helix segments. Residues that are ligands to the manganese are boxed in blue.

The crystal structure of MnSOD-3 solved at 293 K was determined by molecular replacement using the program *Phaser* (Read, 2001 ▶) with the human MnSOD enzyme structure (PDB code 1n0j; Borgstahl *et al.*, 1992 ▶) as the search model (63.6% sequence identity). After initial rounds of rigid-body and restrained refinement using *REFMAC*5 (Murshudov *et al.*, 1997 ▶), the polypeptide chain was mutated to the MnSOD-3 residues and checked against both 2*F*_o_ − *F*_c_ and *F*_o_ − *F*_c_ maps using the program *Coot* (Emsley & Cowtan, 2004 ▶). There were two deletion regions compared with the human model: one that was missing Gly87 and the other Gly147, Thr148 and Thr149 (Fig. 1 ▶). Residues around these regions that did not fit well to the map were removed (85–91 and 147–151; MnSOD-3 numbering) to prevent bias in map generation. Model building and refinement were carried out using *Coot* and *REFMAC*5, allowing the deleted regions to be rebuilt.

The structure of MnSOD-3 solved at 100 K was determined at 1.7 Å resolution from the 293 K MnSOD-3 structure by difference Fourier. Model building and refinement were carried out using *Coot* and *REFMAC*5, allowing 420 water molecules and one malonate ion to be rebuilt, with the *R*_free_ set carried over from the 293 K structure. Noncrystallographic symmetry averaging was not used during structural refinement. In the later stages of refinement, TLS parameters (Winn *et al.*, 2001 ▶) based on a single-group TLS model for each monomer were calculated using the TLS Motion Determination server (http://skuld.bmsc.washington.edu/~tlsmd/) and refined in *REFMAC*5. The refined models of MnSOD-3 at both 293 and 100 K each contain two protein chains in the asymmetric unit, comprising all amino acids in the sequence. The final structure of MnSOD-3 was refined to *R* = 21.6% and *R*_free_ = 26.2% at 293 K and *R* = 18.9% and *R*_free_ = 22.6% at 100 K.

The structure of MnSOD-2 was determined at 1.8 Å resolution from the 100 K MnSOD-3 structure by difference Fourier. Model building and refinement were carried out using *Coot* and *REFMAC*5. Noncrystallographic symmetry averaging was not used during structural refinement. TLS parameters based on a single-group TLS model for each monomer (calculated from the TLS Motion Determination server) were refined at later stages of refinement. The final structure of MnSOD-2 was refined to *R* = 16.9% and *R*_free_ = 20.1%.

## 3. Results and discussion   

Although MnSOD-2 and MnSOD-3 were overexpressed in *E. coli* without an N-terminal histidine tag, they each purified reasonably well using MCAC. A single-column procedure resulted in 66% purification for MnSOD-2 and 79% purification for MnSOD-3 as estimated by SDS–PAGE densitometry. A second MCAC purification yielded MnSOD-2 to 70% purity, while further ion exchange of MnSOD-3 using CM-52 gave a sample that was 98% pure. In a final step, gel-filtration chromatography resulted in 95% pure MnSOD-2 and 99% pure MnSOD-3 (results not shown). The subunit molecular weights of the purified proteins were measured by mass spectrometry and revealed that each of the MnSOD proteins had retained its N-­terminal methionine residue. Gel-filtration chromatography indicated that the pure proteins were tetrameric, a result that was confirmed by analytical ultracentrifugation (results not shown). Specific activity was estimated for MnSOD-2 as 3622 ± 80 U mg^−1^ per manganese and for MnSOD-3 as 3261 ± 26 U mg^−1^ per manganese; the proteins were 60 and 100% metallated with mangan­ese, respectively. These activities are comparable with that of the MnSOD from *E. coli* under the same conditions.

Seven structures of MnSODs have been reported, three of which are tetrameric [human MnSOD (Borgstahl *et al.*, 1992 ▶), *Thermus thermophilus* MnSOD (Ludwig *et al.*, 1991 ▶) and *Aspergillus fumigatus* MnSOD (Fluckiger *et al.*, 2002 ▶)], while the others are dimeric [*Escherichia coli* MnSOD (Edwards *et al.*, 1998 ▶), *Bacillus stearo­thermophilus* MnSOD (Parker & Blake, 1988 ▶), *Bacillus anthracis* MnSOD (Boucher *et al.*, 2005 ▶), *Deinococcus radiodurans* MnSOD (Dennis *et al.*, 2006 ▶) and cyanobacterium MnSOD (Atzenhofer *et al.*, 2002 ▶)]. All the structures share a common overall fold with the iron-containing enzymes. The *C. elegans* MnSODs are structurally similar to the human enzyme; superposition of all the C^α^ atoms of MnSOD-2 and MnSOD-3 with human SOD gives root-mean-square-deviations of 0.5 and 0.8 Å, respectively. One chain (chain *C*) in each SOD asymmetric unit was found to contain electron density corresponding to the N-terminal methionine residue. Two *cis*-prolines (Pro16) are present in each chain in common with other MnSODs. Two residues, Asn141 and Lys169 (MnSOD-2 numbering), have torsion angles in less favourable regions of the Ramachandran diagram (Table 1 ▶). Asn141 adopts ϕ and ψ angles of 52° and −125°, respectively, such that its carbonyl group lies parallel to the indole ring of Trp160 with the oxygen forming a hydrogen bond to N^∊^. Lys169 adopts ϕ and ψ angles of 54° and −133°, with its carbonyl group packing parallel to the Tyr165 side-chain ring.

**Figure 2:**
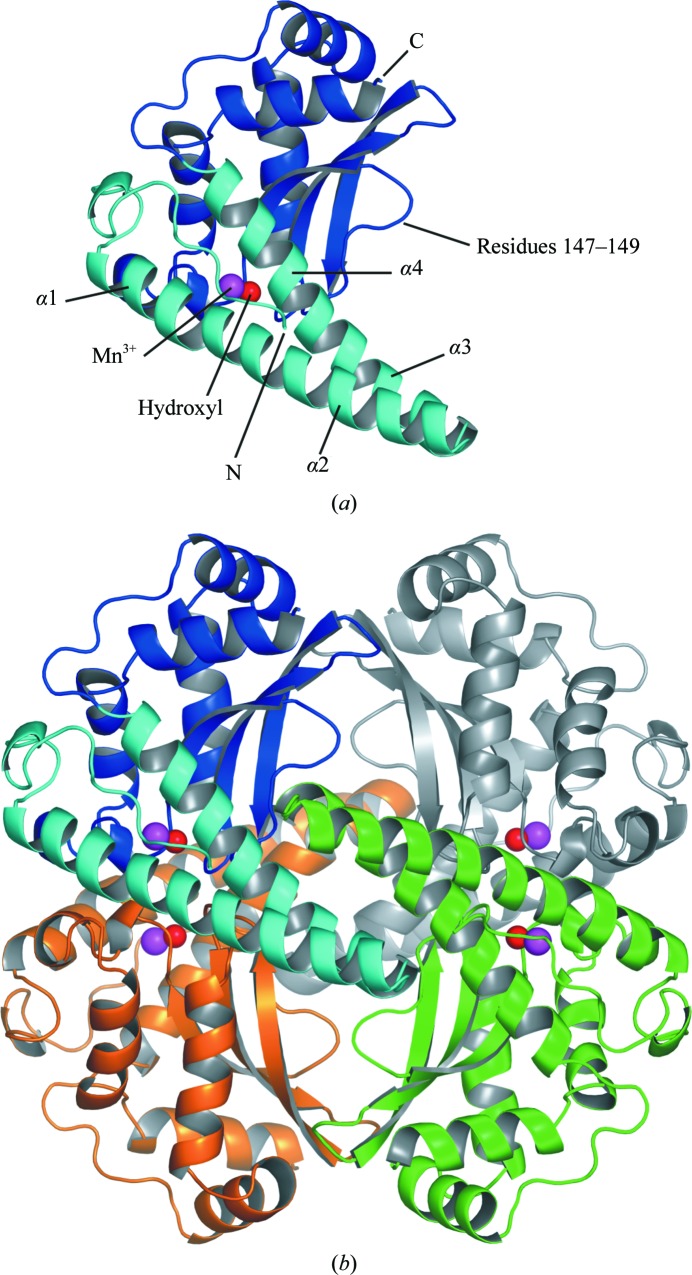
(*a*) Ribbon diagram of the structure of one MnSOD-3 subunit (PDB code 3dc5). The N- and C-domains are shown in light and dark blue, respectively, with the N- and C-termini indicated. Although the N-terminal domain appears to consist of two long helices, each of these is disrupted by a single additional residue (positions 29 and 60, respectively) that bulges out, with the main-chain carbonyl excluded from the regular hydrogen-bonding pattern. The first long helix is therefore comprised of two turns of α1 followed by six turns of α2, while the second has two turns of α3 followed by five turns of α4. α2 and α3 are linked by a hairpin and form a four-helix bundle at the tetramer interface. (*b*) Ribbon diagram of the MnSOD-3 tetramer colored by subunit. The blue subunit is in the same orientation shown in (*a*). The two four-helix bundles of the tetramer interface can be clearly seen centered in the figure. The manganese and hydroxyl ions are also shown in magenta and red, respectively. This figure was produced using *PyMOL* (DeLano, 2008 ▶)

**Figure 3:**
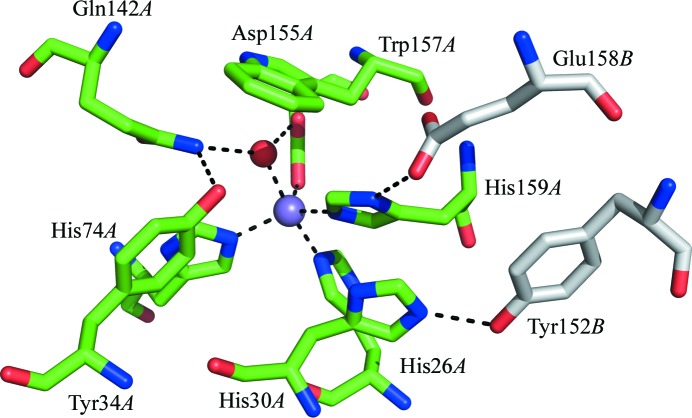
The structure of the active site of MnSOD-3 showing the hydrogen-bonding network viewed from the approximate direction of substrate access. The manganese and the hydroxyl ions are shown as magenta and red spheres, respectively. Residues from different subunits, which form a dimer, are colored as in Fig. 2 ▶(*b*). This figure was produced using *PyMOL* (DeLano, 2008 ▶).

The monomeric fold of mononuclear SODs can be divided into two domains: an α-hairpin N-domain and an α/β C-domain (Fig. 2 ▶). Similar to other reported MnSODs, the tetrameric quaternary structure in MnSOD-2 and MnSOD-3 is accomplished through interactions of the N-domain α-hairpin (Fig. 2 ▶). The ligands of the manganese ion are contributed by residues from each domain: one from each of the α-helices of the N-domain (His26 and His74) and two from the C-domain (Asp158 and His162; MnSOD-2 numbering; Fig. 1 ▶). Metal-coordination bond lengths are given in Table 2 ▶. As seen in other SODs, the metal also binds a water or hydroxyl molecule, forming a five-coordinate trigonal bipyramidal configuration (Fig. 3 ▶). Important residues involved in the hydrogen-bonding network of the active site are shown in Fig. 3 ▶ and include the metal-coordinating residues. Substrate access to the metal is controlled by residues from both subunits (not shown), while two residues from the other subunit forming the dimer contribute to the stability of the active site (Fig. 3 ▶). One of these residues, Glu161 (MnSOD-2 numbering), is highly conserved and may contribute to intersubunit communication during catalysis. MnSOD-3 has a deletion of three amino acids (Ala147–Thr149; Fig. 2 ▶ *a*) compared with other MnSODs apart than that from the cyanobacterium *Anabaena variabilis* (Atzenhofer *et al.*, 2002 ▶). This deletion results in the loss of an α-helical region (α7; Fig. 1 ▶) that would lie between the two chains of the dimer, packing next to α3 of the opposite chain. The major difference between dimeric and tetrameric MnSODs is in the configuration of the α-hairpin of the N-­domain (α2 –α3; Fig. 2 ▶ *a*). In tetrameric MnSODs this remains in an extended conformation allowing two subunits to form a four-helix bundle (as seen in Fig. 2 ▶), thus making a dimer of dimers in the quaternary structure. Characterization of *C. elegans* SODs will lend support to the use of this multicellular organism in model studies of oxidative stress and aging. These structures will also aid in the analysis of the kinetics of SOD activity of these important metallo­enzymes.

## Supplementary Material

PDB reference: manganese superoxide dismutase MnSOD-2, 3dc6, r3dc6sf

PDB reference: MnSOD-3, 3dc5, r3dc5sf

## Figures and Tables

**Table 1:** Crystallographic summary of the structures of MnSOD-2 and MnSOD-3 Values in parentheses are for the outermost shell.

	SOD-2 (100K)	SOD-3 (100K)	SOD-3 (293K)
Resolution range ()	69.81.8 (1.91.8)	44.11.7 (1.791.7)	57.641.77 (1.871.77)
Space group	*P*4_1_2_1_2	*P*4_1_2_1_2	*P*4_1_2_1_2
Unit-cell parameters ()	*a* = *b* = 81.0, *c* = 137.4	*a* = *b* = 81.8, *c* = 136.0	*a* = *b* = 81.5, *c* = 138.0
No. of observed reflections	172926	181809	190572
No. of unique reflections	41573	49346	45989
Redundancy	4.2 (4.2)	3.7 (3.6)	4.1 (4.0)
Completeness (%)	97.2 (98.9)	96.2 (95.8)	99.9 (99.9)
*I*/(*I*)	8.7 (5.1)	5.0 (2.1)	8.6 (2.3)
*R*_merge_ † (%)	5.6 (14.4)	7.6 (36.4)	6.2 (33.8)
Refinement and model statistics			
Resolution range for refinement ()	43.991.8	44.101.7	70.191.77
*R* factor (%)	16.9‡	18.9	21.6
*R*_free_ § (%)	20.1‡	22.6	26.2
No. of protein non-H atoms	3170	3138	3138
No. of water molecules	448	420	298
No. of manganese ions	2	2	2
No. of sulfate atoms	15	0	0
No. of malonate-ion atoms	0	7	0
R.m.s.d. bond lengths¶ ()	0.009	0.013	0.015
R.m.s.d. bond angles¶ ()	1.1	1.4	1.4
*B* factors (^2^)			
Overall	16	19	23
Protein	14	18	22
Water	29	32	26
Manganese	14	15	15
Sulfate	33	0	0
Malonate	0	26	0
Ramachandran analysis†† (%)			
Residues in most favoured regions	92.2	91.5	92.7
Residues in additional allowed regions	6.7	7.3	6.1
Residues in generously allowed regions	1.2	1.2	1.2

† *R*_merge_ = 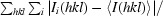 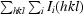.

‡ The *R*_free_ set from the MnSOD-3 (100K) refinement was not transferred to the MnSOD-2 structure, leading to a slightly lower difference between *R* and *R*_free_, but they both fell normally during rebuilding of side chains that differ between the two homologous structures.

§ *R*_free_ was calculated using 5% of the reflections that were set aside randomly.

¶ Based on the ideal geometry values of Engh Huber (1991 ▶).

†† Ramachandran analysis using *PROCHECK* (Laskowski *et al.*, 1993 ▶).

**Table 2:** Mn^III^-coordination distances, given as determined from independent subunits of the asymmetric unit

	Bond length† ()					
	MnSOD-2 (100K)		MnSOD-3 (100K)		Human (100K)‡	
Mn-coordinating residue	Subunit *A*	Subunit *C*	Subunit *A*	Subunit *C*	Subunit *A*	Subunit *B* §
His26	2.2	2.2	2.2	2.2	2.2	2.4
His74	2.2	2.2	2.2	2.2	2.3	2.2
Asp158 (MnSOD-2)/Asp155 (MnSOD-3)/Asp159 (human)	2.0	2.0	2.1	2.1	2.0	2.0
His162 (MnSOD-2)/His159 (MnSOD-3)/His163 (human)	2.2	2.2	2.2	2.1	2.1	2.2
Hydroxyl	2.0	2.1	2.3	2.3	2.2	2.0

† The error associated with the bond lengths is 0.2 as calculated from the Cruickshank diffraction-component precision index (Cruickshank, 1999 ▶).

‡ Human manganese superoxide dismutase (PDB code 1luv; Hearn *et al.*, 2003 ▶).

§ Subunit *B* in human MnSOD is equivalent to subunit *C* in MnSOD-2 and MnSOD-3.
